# Juvenile Moyamoya and Craniosynostosis in a Child with Deletion 1p32p31: Expanding the Clinical Spectrum of 1p32p31 Deletion Syndrome and a Review of the Literature

**DOI:** 10.3390/ijms18091998

**Published:** 2017-09-17

**Authors:** Paolo Prontera, Daniela Rogaia, Amedea Mencarelli, Valentina Ottaviani, Ester Sallicandro, Giorgio Guercini, Susanna Esposito, Anna Bersano, Giuseppe Merla, Gabriela Stangoni

**Affiliations:** 1 Medical Genetics Unit, S. Maria della Misericordia Hospital, University of Perugia, 06123 Perugia, Italy; paolo.prontera@ospedale.perugia.it (P.P.); daniela.rogaia@unipg.it (D.R.); amedea.mencarelli@unipg.it (A.M.); ester_salli@libero.it (E.S.); gabriela.stangoni@ospedale.perugia.it (G.S.); 2 Medical Genetics Unit, IRCCS Casa Sollievo della Sofferenza Hospital, San Giovanni Rotondo, 71013 Foggia, Italy; ottaviani.va@tiscali.it (V.O.); (g.merla@operapadrepio.it (G.M.); 3 Neuroradiology Department, S. Maria della Misericordia Hospital, University of Perugia, 06123 Perugia, Italy; giorgio.guercini@ospedale.perugia.it; 4 Pediatric Clinic, Department of Surgical and Biomedical Sciences, Università degli Studi di Perugia, Piazza Menghini 1, 06129 Perugia, Italy; 5 Cerebrovascular Unit, IRCCS Istituto Neurologico Carlo Besta, 20133 Milan, Italy; anna.bersano@istituto-besta.it

**Keywords:** 1p32p31 deletion, moyamoya syndrome, craniosynostosis, *FOXD3*, *FOXC1*, *FOX* genes

## Abstract

Moyamoya angiopathy (MA) is a rare cerebrovascular disorder characterised by the progressive occlusion of the internal carotid artery. Its aetiology is uncertain, but a genetic background seems likely, given the high MA familial rate. To investigate the aetiology of craniosynostosis and juvenile moyamoya in a 14-year-old male patient, we performed an array-comparative genomic hybridisation revealing a de novo interstitial deletion of 8.5 Mb in chromosome region 1p32p31. The deletion involved 34 protein coding genes, including *NF1A*, whose haploinsufficiency is indicated as being mainly responsible for the 1p32-p31 chromosome deletion syndrome phenotype (OMIM 613735). Our patient also has a deleted *FOXD3* of the *FOX* gene family of transcription factors, which plays an important role in neural crest cell growth and differentiation. As the murine *FOXD3*^−/−^ model shows craniofacial anomalies and abnormal common carotid artery morphology, it can be hypothesised that *FOXD3* is involved in the pathogenesis of the craniofacial and vascular defects observed in our patient. In support of our assumption, we found in the literature another patient with a syndromic form of MA who had a deletion involving another *FOX* gene (*FOXC1*). In addition to describing the clinical history of our patient, we have reviewed all of the available literature concerning other patients with a 1p32p31 deletion, including cases from the Decipher database, and we have also reviewed the genetic disorders associated with MA, which is a useful guide for the diagnosis of syndromic form of MA.

## 1. Introduction

Moyamoya angiopathy (MA) is a cerebrovascular occlusive disorder characterised by bilateral progressive stenoses of the terminal portions of the internal carotid and the proximal anterior and middle cerebral arteries, in addition to the formation of compensatory collateral (moyamoya) vessels at the base of the brain [1]. Associations between these vascular conditions and other disease entities such as atherosclerosis, autoimmune disease, meningitis, brain tumours and genetic disorders define “moyamoya syndrome” (MMS) [2] (Table 1), whereas “moyamoya disease” (MMD) indicates the most common, generally idiopathic or isolated form.

The aetiology of moyamoya is still unknown, but familial aggregation and association studies suggest a genetic background, although the studies carried out so far have not revealed any significant locus associated with moyamoya other than *RNF213*, which seems to confer susceptibility to the disease in Asian countries [6,20,21]. Rare missense mutations in this gene have been significantly associated to MA European patients, particularly in childhood-onset and familial cases [2]. However, recently familial cases of MA and stereotyped facial dysmorphisms and early-onset achalasia, carrying respectively mutations in *BRCC3* deubiquitinase and in *GUCY1A3*, the gene encoding the major nitric oxide receptor in vascular smooth muscle cells (vSMCs), have been reported [16,22]. We here describe the case of a 14-year-old patient with an intellectual disability (ID), craniosynostosis and juvenile moyamoya, carrying an 8.5 Mb de novo deletion in the chromosomal region 1p32.2p31.3, a newly recognized genomic disorder to be added to the growing number of genetic and genomic syndromes associated with MA.

## 2. Case Report

### Patient Presentation

Informed consent to the study and the publication of the results was given by the proband’s parents.

**Figure 1:**
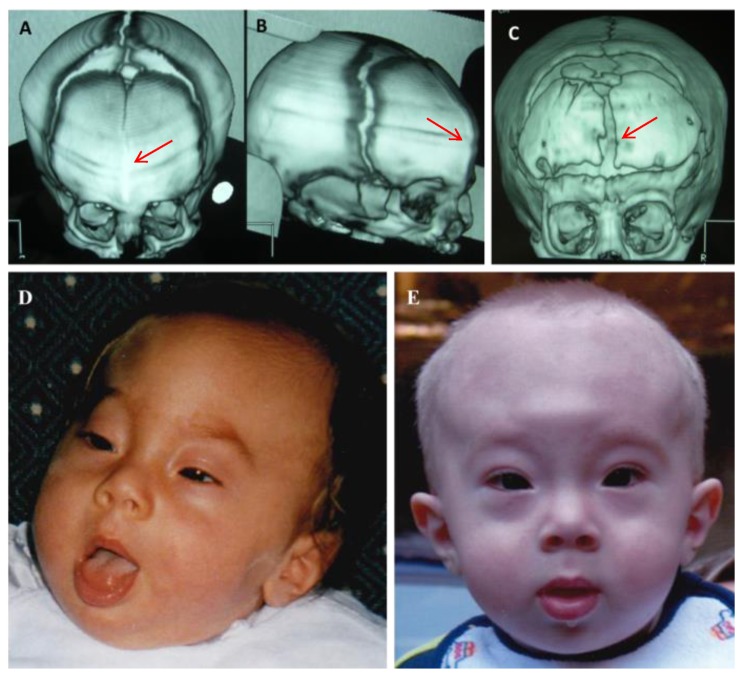
Computed tomography (CT) scan of the cranium with helical and 3D reconstruction. (**A**,**B**) One month old: skull morphology appears triangular; the metopic suture is already calcified in its middle-lower portion (red arrows), and gives the skull a trigonocephalic aspect; (**C**) Eleven months old: results of bilateral frontal bone craniotomy with removal of the median hyperostotic ridge (red arrow); (**D**) Patient one month old, showing a high forehead, a prominent metopic suture, bitemporal narrowing, up-slanting palpebral fissures, and anteverted nares; (**E**) Patient eight months old, after the craniotomy, has a wider bitemporal space and less pronounced up-slanting palpebral fissures.

**Figure 2:**
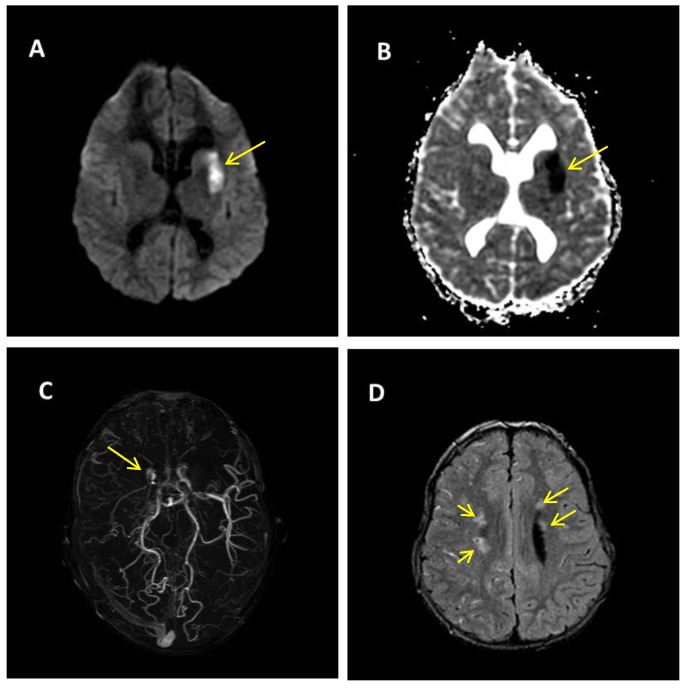
Brain magnetic resonance imaging (MRI) at the age of 10 years, after the first TI sequence. (**A**,**B**) Diffusion weighted imaging (**A**) and diffusion coefficient map (**B**) show a lesion of the left lenticular nucleus of the anterior part of the internal capsula, and the head and body of the lateral nucleus caudatus characterised by reduced diffusion due to an acute ischemic lesion (yellow arrows). (**C**) Angio-TOF MRI showing the stenosis of the right carotid siphon apex (yellow arrow) associated with poorly visualised flow of the ipsilateral middle cerebral artery, stenosis of the left carotid siphon apex, and a stretch of the M1 ipsilateral middle cerebral artery; (**D**) Fluid attenuation inversion recovery (FLAIR) sequence: small areas of signal hyperintensity at the semioval centres bilaterally (yellow arrows), most marked on the right side.

The 14-year-old boy was born to healthy non-consanguineous parents. At birth, he showed complex craniofacial abnormalities (Figure 1D), and 3D cerebral computed tomography (CT) revealed a hyperostotic metopic suture, bilateral aplasia of the frontal bones, a hypoplastic supraorbital ridge (Figure 1A,B); additionally, magnetic resonance imaging (MRI) revealed a cyst in the septum pellucidum associated with mild ventricular dilatation. At the age of six months, he underwent neurosurgery and plastic reconstruction, including bi-frontal craniotomy, orbital osteotomies, and the remodelling of the frontal skull vault and the supraorbital margins (Figure 1C,E). The post-operative course was uneventful. His motor development was normal (walking at the age of 12 months) but a speech delay required speech therapy. The results of a neuropsychiatric evaluation when he was four years old indicated mild intellectual disability (IQ 70). Follow-up MRI when he was eight years old confirmed the presence of the cyst in the septum pellucidum and revealed the presence of multiple punctate areas of altered signal, which were more pronounced in the right hemisphere (Figure 2D), and an atrophic aspect of the corpus callosum. At the age of 10, he experienced an episode of right-sided weakness and a subsequent brain MRI showed acute ischemic stroke of the basal ganglia bilaterally, mainly on the left side (Figure 2A,B).

One year later, during a febrile episode, he experienced a tonic-clonic seizure involving the right upper limb. He repeated brain MRI associated with cerebral angiography, which failed to visualise the right internal carotid artery but showed a severe stenosis of the left internal carotid artery in association with dilated collateral middle cerebral artery (MCA) vessels having the typical “puff of smoke” aspect (Figure 2C). A CT scan with acetazolamide showed a marked reduction in the time of transit of the contrast medium in bi-hemispheric cortical space that suggested reduced perfusion in both the Sylvian and the anterior circulation territories of the carotids bilaterally, with no signs of reserve. On the basis of the established criteria [23], the neuroradiological findings were diagnostic of MA.

At the age of 11, the patient underwent right encephalomiosynangiosis surgery, which was followed by an improvement in his school performance and the absence of any further episodes of stroke or stroke-like activity. No other surgical or pharmacological interventions have been carried out since.

**Figure 3:**
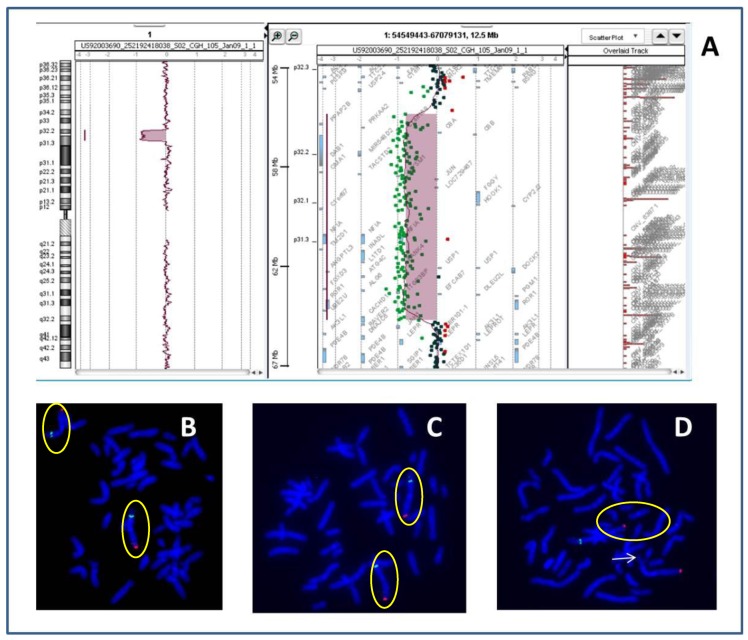
(**A**) Partial result of an array-comparative genomic hybridisation (a-CGH) in the patient showing on the left the general profile of chromosome 1, and on the right the deleted 1p32p31 region. The genes included in the deleted region are: *PPAP2B*; *PRKAA2*; *C1orf168*; *C8A*; *C8B*; *DAB1*; *MIR548D2*; *OMA1*; *TACSTD2*; *MYSM1*; *JUN*; *LOC729467*; *FGGY*; *HOOK1*; *CYP2J2*; *C1orf87*; *NFIA*; *TM2D1*; *INADL*; *L1TD1*; *KANK4*; *USP1*; *DOCK7*; *ANGPTL3*; *ATG4C*; *FOXD3*; *ALG6*; *ITGB3BP*; *EFCAB7*; *DLEU2L*; *PGM1 ROR1 UBE2U*, and; *CACHD1*; (**B**,**C**) Results of Fluorescence in Situ Hybridization (FISH) experiments conducted on metaphases obtained from lymphocytes cultures of peripheral blood of the mother (**B**), the father (**C**) and the patient (**D**), showing normal red (specific probe for the subtelomeric 1q region) and green (specific probe for the 1p32 region) signals in the parents (target chromosomes encircled by yellow ovals), indicating the absence of the deletion of 1p32 (**B**,**C**), that instead is present in the patient (**D**), where the green signal is absent on the chromosome 1p32, indicated by the white arrow, the normal chromosome is also encircled by a yellow oval.

At the age of 12, the patient was evaluated by a medical geneticist who suggested an array-comparative genomic hybridisation (a-CGH) analysis, which was made on DNA extracted from peripheral blood of the patient using an Agilent Human Genome CGH Microarray Kit 60K (Agilent Technologies, Santa Clara, California, USA) as previously described [24]. The analysis revealed an 8.5 Mb deletion in chromosomal region 1p32.2p31.3 involving 34 genes, including the OMIM-annotated genes *C8A*, *C8B*, *TACSTD2*, *ANGPTL3*, *FOXD3*, *ALG6* and *PGM1* (arr 1p32.2–p31.3(56,591,330-65,098,261)x1) (Genome Assembly, February 2009 GRCh37, hg19) (Figure 3A). Fluorescent in situ hybridisation (FISH) analyses of both parents and the proband using the RP11-37M11 BAC clone (1p32.1-start 60,166,741–60,309,110 end; Hg19) (BlueFish Blue Genome, Cambridge, UK) as previously described [25] established the de novo origin of the deletion (Figure 3B–D).

## 3. Discussion

We here describe the first moyamoya patient carrying a deletion at 1p32p31. The rarely reported deletions at 1p32p31 are mainly associated with brain malformations (absence or hypoplasia of the corpus callosum, ventriculomegaly, and macrocephaly), urinary tract defects (vescicouretral reflux, urinary incontinence), facial dysmorphisms, and developmental delay. This condition has recently been annotated as “1p32p31 chromosome deletion syndrome” (OMIM 613735), and it has been suggested that the phenotype is the result of haploinsufficiency of the *NFIA* gene [26]. Only eight patients have been reported in the literature [27], but we found 11 further patients with 1p32p31 deletions involving the *NFIA* gene recorded in the Decipher v9.1 database (Wellcome Trust Sanger Institute: http://decipher.sanger.ac.uk) [28]. Of the 20 patients with 1p32p31 deletions, 10 (50%) show the involvement of both the *NFIA* and *FOXD3* genes; only one patient (Decipher database ID: 252422), for whom no clinical information is available, has the deletion of *FOXD3* but not *NFIA*. The variability in the extent of these deletions is probably responsible for the phenotypic variability of the patients but consideration of all of the reported cases, including our patient, makes it possible to define a pattern of recurrent clinical signs and symptoms (Table 2).

As MA may be due to the haploinsufficiency of a gene in the 1p32p31 region, and this could be a starting point for future investigations of its genetics aspects, we carefully evaluated all of the genes deleted in our patient. In particular the *FOXD3* gene (Forkhead box D3-OMIM 611539) aroused our interest because of its function and its analogy to another *FOX* gene (*FOXC1*) that was found to be deleted in another seven-year-old patient with juvenile moyamoya, ID and craniofacial dysmorphisms [5].

The *FOX* gene family consists of a large number of genes encoding for transcription factors that play a critical role in the differentiation and development of neural crest cells (NCCs). *FOXD3* is expressed in the epiblast during early embryogenesis and later in NCCs. Many studies of various animal models (early chicken embryos, mouse embryos, zebrafish) have highlighted the critical role of *FOXD3* neural crest (NC) development as it participates in segregating the NC lineage from the neural epithelium. In mouse embryos, *FOXD3* is expressed in pre-migratory and migratory NCCs, and is required for the maintenance of multipotent NC progenitors by self-renewing and repressing differentiation [29,30]. Notably, cephalic NCCs migrate to various regions in the head and neck where they contribute to the development of structures as diverse as the anterior skull base, the walls of the craniofacial arteries, the forebrain, and the face [31], which is in line with the pattern of craniofacial and vascular malformations shown by *FOXD3* knock-out mice.

The *FOXD3*^tm2Lby^/*FOXD3*^tm3Lby^ murine model (Mouse Genome Informatics: http://www.informatics.jax.org) [32] presents a morphologically abnormal neurocranium due to the reduced growth rate of cranial bones. It is widely known that craniosynostosis is characterised by a primary abnormality of skull growth, with the premature fusion of the cranial sutures that appear as a result of the difference in the rate of growth between the skull and developing brain. Furthermore, this animal model also shows a morphologically abnormal common carotid artery from which the abnormal internal carotid arteries of moyamoya arise. These findings suggest the possible role of *FOXD3* haploinsufficiency in the pathogenesis of the craniosynostosis and MA of our patient but, as this is not supported by other case reports of patients with *FOXD3* deletions, we can hypothesise the reduced penetrance of a single gene defect (clinical variability is very frequent when a transcription factor is involved in the pathogenesis of human diseases) [33], or a polygenic or multifactorial origin of the vascular defects. Intriguingly, NC disease could explain the association between craniosynostosis and MA in our patient and others as craniosynostosis is one of the most frequent comorbidities found in patients with syndromic moyamoya [34], and could also be helpful in clarifying the presence of the segmental vascular alterations.

## 4. Conclusions

Patients with 1p32p31 deletion do not show patognomonic features, but the general clinical presentation (developmental delay, facial dysmorphism, brain and/or genito-urinary malformations) is suggestive of a genomic disorder, prompting to the diagnosis by a-CGH or single nucleotide polymorphism (SNP)-array analyses. Our case expands the clinical spectrum of the diseases associated with 1p32-p31 deletions, further underlining the importance of genomic analyses of patients with MMS, and contextually suggests adding mutations of *FOX* gene family to the heterogeneous genetic causes of MA.

## Figures and Tables

**Table 1:** Chromosomic, genomic and monogenic disorders associated with moyamoya angiopathy (MA).

**Chromosomic Disorders**	**Chromosome Involved**
Down syndrome (complete or mosaic form) [3]	21
Turner syndrome (complete or mosaic form) [4]	X
**Genomic Disorders**	**Gene/s Involved**
6p25.3-p23 del/dup and 12q24.32-qter dup [5]	On 6p region: *IRF4* and other 51 OMIM genes including *FOXC1*; On 12q region: *22* OMIM genes not associated with genetic disorders.
15q13.3 duplication (Decipher ID: 263336) [2,6]	*CHRNA7*, *OTUD7A*
Xq28 deletion [7]	*F8* (exon 1-6), *FUNDC2*, *MTCP1NB*, *MTCP1*, *BRCC3*
Smith-Magenis syndrome (del 17p11.2-p13.1) [8]	More than 25 genes including: *RAI1*; *MED9*; *RASD1*; *FLCN*; *PMP22; COX10*; *ELAC2*; *ZNF18*; *MYH1*
Trisomy 12p [9]	Genes included in the region of the rearrangement: 46, XX, rec(12)dup(12p)inv(12)(p11.2q24.3)mat
1p32p31 deletion (present report)	OMIM genes included in the region of the rearrangement: *C8A*; *C8B*; *TACSTD2*; *ANGPTL3*; *FOXD3*; *ALG6*; *PGM1*
**Monogenic Disorders**	**Gene/s Involved**
Type 1 Neurofibromatosis [10]	*NF1*
Noonan syndrome [11]	*PTPN11*, *SOS1*, *RAF1* and, more rarely: *KRAS*; *NRAS*; *BRAF,* and; *MAP2K1*
Costello syndrome [12]	*HRAS*
Alagille syndrome [13]	*JAG1*, *NOTCH2*
Marfan syndrome [14]	*FBN1*
Sickle cell disease [15]	*HBB*
Moyamoya disease-6 with achalasia [16]	*GUCY1A3*
*SAMHD1*-related disorders [17]	*SAMHD1*
MOPD2/Majewski syndrome [18]	*PCNT*
Seckel syndrome (microcephalic primordial dwarfism) [19]	*ATR*, *RBBP8*, *CENPJ*, *CEP152*, *CEP63*, *NIN*

**Table 2:** Clinical features in patients with 1p32-p31 deletion involving *NFIA* gene.

Clinical Features	Present Case	Frequency on Reported Cases (Literature, Decipher, Present Case)	Total	References
Brain malformations	+	12/20	60%	Decipher ID: 288170; Literature: [16,17,18,19,20,21]
Corpus callosum defects	+	11/20	55%	Decipher ID: 251391-4638; Literature: [16,17,18,19,20,21]
Facial dysmorphisms	+	9/20	45%	Decipher ID: 264827-285848; Literature: [16,17,18,19,20,21]
Genito-urinary defects	+	7/20	35%	Literature: [16,18,19,20,21]
Developmental delay	−	6/20	30%	Decipher ID: 264827-285848; Literature: [16,17,19,20]
Intellectual disability	+	5/20	25%	Decipher ID: 251391-288170-4638
Hypotonia	+	5/20	25%	Decipher ID: 285848; Literature: [16,17,19,20]
Language disabilities	+	3/20	15%	Decipher ID: 276512
Craniosynostosis	+	2/20	10%	Decipher ID: 251391
Moyamoya disease	+	1/20	5%	-
